# Ganglioside GM1 Alleviates Propofol-Induced Pyroptosis in the Hippocampus of Developing Rats via the PI3K/AKT/NF-κB Signaling Cascade

**DOI:** 10.3390/ijms252312662

**Published:** 2024-11-25

**Authors:** Zhiheng Zhang, Shan Du, Xinzhang Chen, Di Qiu, Siyao Li, Lin Han, Hui Bai, Ruifeng Gao

**Affiliations:** 1 College of Veterinary Medicine, Inner Mongolia Agricultural University, Hohhot 010018, China; 2 Key Laboratory of Clinical Diagnosis and Treatment Techniques for Animal Disease, Ministry of Agriculture, Inner Mongolia Agricultural University, Hohhot 010018, China; 3 College of Veterinary Medicine, Northeast Agricultural University, Harbin 150030, China

**Keywords:** propofol, GM1, neuroinflammation, PI3K/AKT/NF-κB signaling, pyroptosis, developing rats

## Abstract

In pediatric and intensive care units, propofol is widely used for general anesthesia and sedation procedures as a short-acting anesthetic. Multiple studies have revealed that propofol causes hippocampal injury and cognitive dysfunction in developing animals. As is known, GM1, a type of ganglioside, plays a crucial role in promoting nervous system development. Consequently, this study explored whether GM1 mitigated neurological injury caused by propofol during developmental stages and investigated its underlying mechanisms. Seven-day-old SD rats or PC12 cells were used in this study for histopathological analyses, a Morris water maze test, a lactate dehydrogenase release assay, Western blotting, and an ELISA. Furthermore, LY294002 was employed to explore the potential neuroprotective effect of GM1 via the PI3K/AKT signaling cascade. The results indicated that GM1 exerted a protective effect against hippocampal morphological damage and pyroptosis as well as behavioral abnormalities following propofol exposure by increasing p-PI3K and p-AKT expression while decreasing p-p65 expression in developing rats. Nevertheless, the inhibitor LY294002, which targets the PI3K/AKT cascade, attenuated the beneficial effects of GM1. Our study provides evidence that GM1 confers neuroprotection and attenuates propofol-induced developmental neurotoxicity, potentially involving the PI3K/AKT/NF-κB signaling cascade.

## 1. Introduction

Millions of infants and young children receive general anesthesia (such as propofol) every year for surgical procedures. Propofol is widely used due to its rapid onset, brief duration, and minimal adverse effects during recovery [1,2,3]. After an intravenous injection, propofol rapidly induces central nervous system (CNS) inhibition by regulating N-methyl-D-aspartate (NMDA) and GABA_A_ receptors [4,5]. Accumulating evidence has shown that propofol affects long-term cognitive abilities, causing neurodevelopmental abnormalities in rodents, zebrafish, piglets, and primates [6,7,8,9]. Moreover, propofol-induced neurotoxicity in developing animals has been associated with neuroinflammation, neuronal and oligodendrocyte death, dysregulated neurogenesis, the downregulation of neurotrophic factors, abnormal dendritic development, and mitochondrial damage [10]. Exploring the fundamental mechanisms behind propofol-induced neurotoxic effects may contribute to the development of successful protective or therapeutic approaches.

The characteristics of pyroptosis include the formation of pores in membranes, cell integrity destruction, and a high inflammatory response. Pyroptosis plays a significant role in neurodegeneration and neuroinflammation, which can cause a variety of CNS diseases, including sepsis [11], traumatic brain injury (TBI) [12], Alzheimer’s disease (AD) [13], and epilepsy [14]. Furthermore, the pyroptosis-induced activation of inflammasome complexes has been associated with the progression of a range of CNS disorders, contributing to neurodegenerative ailments and damage to nerve cells, including demyelination and excitotoxicity [15]. Several studies have shown that nuclear factor-κB (NF-κB)-related inflammasome activation is involved in the pathogenesis of general-anesthesia-induced neurotoxicity [16,17]. In addition, PI3K/AKT has been found to inhibit NF-κB (via the phosphorylation of p65) and facilitates the movement of p-p65 into the nucleus [18]; moreover, the PI3K/AKT/NF-κB pathway is vital for general anesthetic neurotoxicity [19]. Further research is still needed to confirm whether this pathway is involved in propofol-induced neurotoxic effects in developing animals.

Gangliosides, present in the cell membrane of all nerve cells, are glycosphingolipids that contain sialic acid residues. These residues are connected to the hydrophobic ceramide tail through glycosidic bonds and serve as anchors for gangliosides on the plasma membrane. Clusters of gangliosides have the capacity to interact with membrane proteins or glycoproteins, thereby modulating cellular signal transduction and intercellular communication [20]. Gangliosides are essential for brain development, and reduced levels lead to neurodegeneration in mammals [21]. As the most abundant ganglioside in mammals, GM1 is highly abundant in the synaptic plasma membrane microdomain of the plasma membrane in neurons [22]. GM1 is crucial for maintaining neuronal development and homeostasis by inhibiting inflammatory responses, excitotoxicity responses, and oxidative stress responses and regulating Ca^2+^ signaling, neurotrophic factor pathways, and mitochondrial function [23]. Nevertheless, the beneficial properties of GM1 against propofol-induced brain damage in developing animals and its specific mechanisms are yet to be elucidated.

In this study, GM1 was used as a neuroprotective agent to explore the regulation mechanisms of propofol-induced hippocampal pyroptosis and developmental neurotoxicity in young rats through the PI3K/AKT/NF-κB signaling pathway. This study investigated the modulatory effects of GM1 on hippocampal pyroptosis and developmental neurotoxicity caused by propofol in developing rats and elucidated its neuroprotective mechanisms.

## 2. Results

### 2.1. Impact of GM1 on Propofol-Induced Hippocampal Injury and Cognitive Deficits in Developing Rats

**Figure 1:**
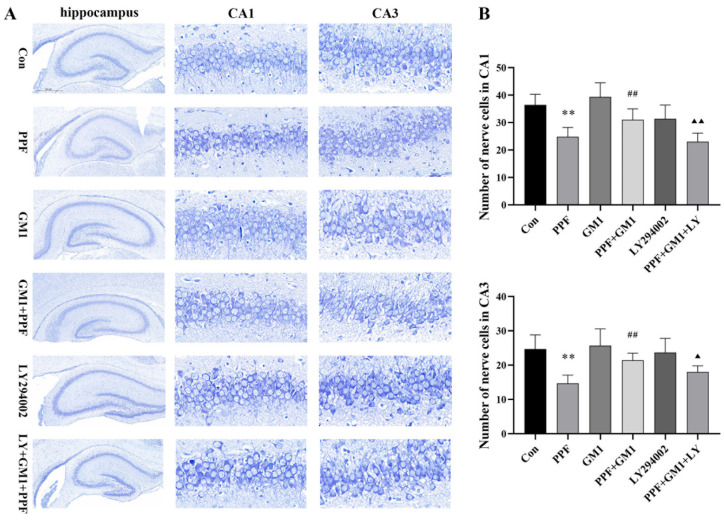
Effect of GM1 on hippocampus morphological injury induced by propofol in young rats. (**A**) The hippocampal sections underwent histological analysis using Nissl staining. The whole hippocampal region was examined at 40× magnification and the CA1 and CA3 regions were observed at 400× magnification. (**B**) Cell counting was conducted in the hippocampal CA1 and CA3 regions (n = 3), where 9 randomly selected fields of view (10^4^ μm^2^ each) were subjected to semi-quantitative analysis to determine cell density (n = 3). ** *p* < 0.01 vs. Con, ^##^ *p* < 0.01 vs. PPF, ^▲^ *p* < 0.05, and ^▲▲^ *p* < 0.01 vs. PPF + GM1.

To determine the effect of GM1 on propofol-related hippocampal damage, Nissl staining, along with a semi-quantitative analysis, was performed on hippocampal regions CA1 and CA3. Our findings revealed that continuous propofol treatment led to a reduction in Nissl bodies, a significant decrease in the nerve cell count (*p* < 0.01), and a disordered arrangement in the CA1 and CA3 regions in the hippocampus of PND 7 rats. However, a pretreatment with GM1 ameliorated propofol-induced reduction or disorganization in the CA1 and CA3 regions. Conversely, LY294002, a PI3K/AKT pathway inhibitor, attenuated the protective effect of GM1 against propofol-related hippocampal damage (Figure 1A,B).

**Figure 2:**
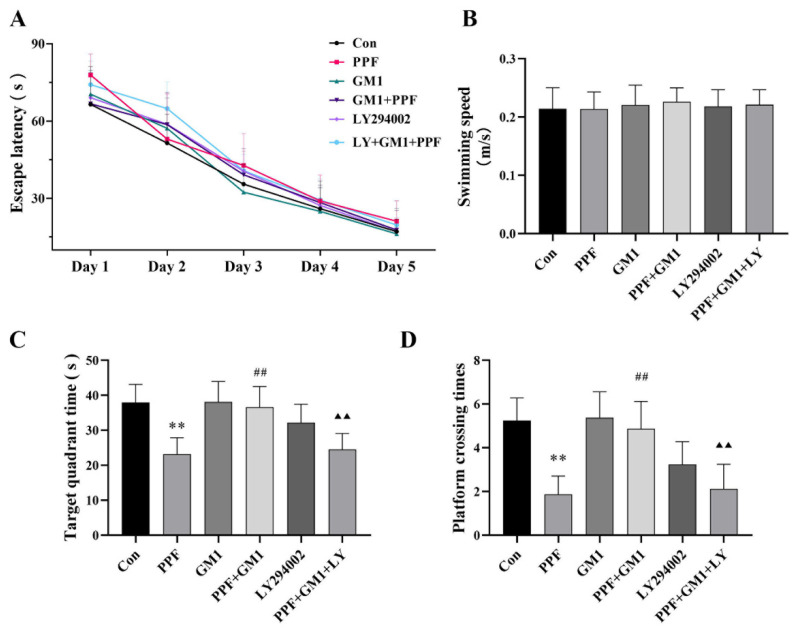
GM1 ameliorates cognitive deficits caused by propofol in young rats. (**A**) Escape latency to reach a platform. (**B**) Mean swimming trail. (**C**) Travel time to target region. (**D**) Time to the target platform (n = 10). ** *p* < 0.01 vs. Con, ^##^ *p* < 0.01 vs. PPF, and ^▲▲^ *p* < 0.01 vs. PPF + GM1.

The MWM test was applied to evaluate the protective effects of GM1 in terms of behavioral impairments in neonatal rats after propofol exposure at PND 30. No significant differences were noted in escape latency or average swimming speeds among the groups (Figure 2A,B). Continuous propofol exposure decreased the time in the target region as well as the number of platform crossings during exploratory trials (*p* < 0.01), while a pretreatment with GM1 significantly alleviated these behavioral effects following continuous propofol exposure. However, LY294002 effectively reversed the impact of GM1 in the LY+ GM1 + PPF group (Figure 2C,D). Collectively, these findings indicated that prolonged propofol exposure led to hippocampal damage and cognitive deficits in developing rats, whereas a GM1 pretreatment offered neuroprotection, potentially involving the PI3K/AKT pathway.

### 2.2. PI3K/AKT/NF-κB Pathway Involved in Propofol-Induced Hippocampal Injury

**Figure 3:**
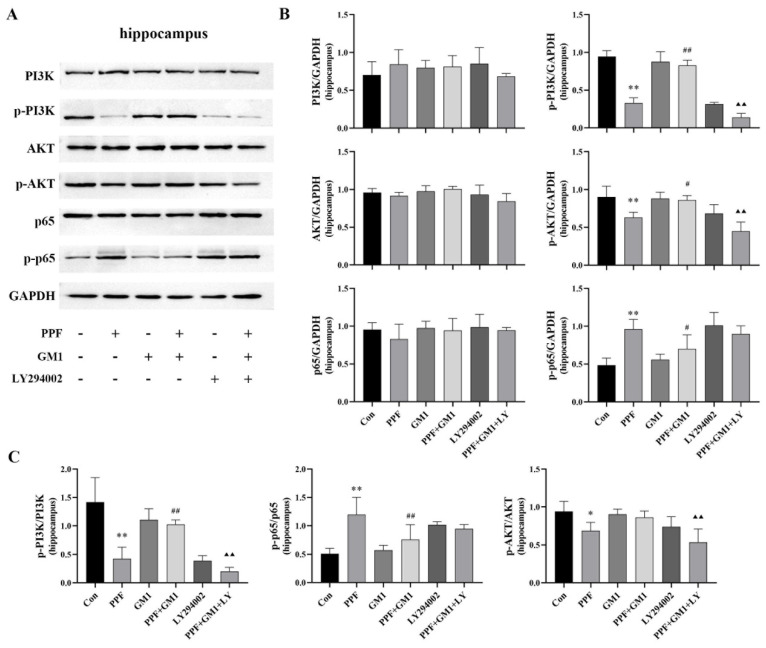
GM1 modulates the PI3K/AKT/NF-κB signaling pathway in the hippocampus of young rats following exposure to propofol. (**A**) Immunoblots and representative images of the PI3K/AKT/NF-κB pathway. (**B**,**C**) Corresponding quantification analysis of PI3K, p-PI3K, AKT, p-AKT, p65, p-p65, p-PI3K/PI3K, p-AKT/AKT, and p-p65/p65 in the hippocampus (n = 3). * *p* < 0.05, ** *p* < 0.01 vs. Con, ^#^ *p* < 0.05, ^##^ *p* < 0.01 vs. PPF, and ^▲▲^ *p* < 0.01 vs. PPF + GM1.

Several studies have demonstrated that the PI3K/AKT/NF-κB pathway is involved in cognitive dysfunction with neuroinflammation [24,25]. To further investigate the potential mechanism of GM1 in neuroprotection, the activation of this signaling pathway in developing rats was assayed at the protein level using Western blotting. No significant changes in the levels of PI3K, AKT, and p65 proteins were observed in the hippocampus among the experimental groups. Our results showed a significant decrease in phosphorylated proteins (p-PI3K and p-AKT) as well as in the ratios of p-PI3K/PI3K and p-AKT/AKT in the hippocampus of developing rats following propofol treatment (*p* < 0.01), along with an increase in p-p65 expression and the p-p65/p65 ratio (Figure 3A–C). Notably, GM1 attenuated the impact of propofol on this pathway; however, following treatment with LY294002, this effect was no longer observed in the GM1 + PPF + LY294002 group. The results indicated that GM1 might exert a neuroprotective effect by activating PI3K/AKT signaling, thus inhibiting NF-κB pathway activity within the hippocampus of developing rats.

### 2.3. GM1 Attenuates Propofol-Induced Pyroptosis and Inflammation of the PI3K/AKT/NF-κB Pathway in the Hippocampus of Developing Rats

**Figure 4:**
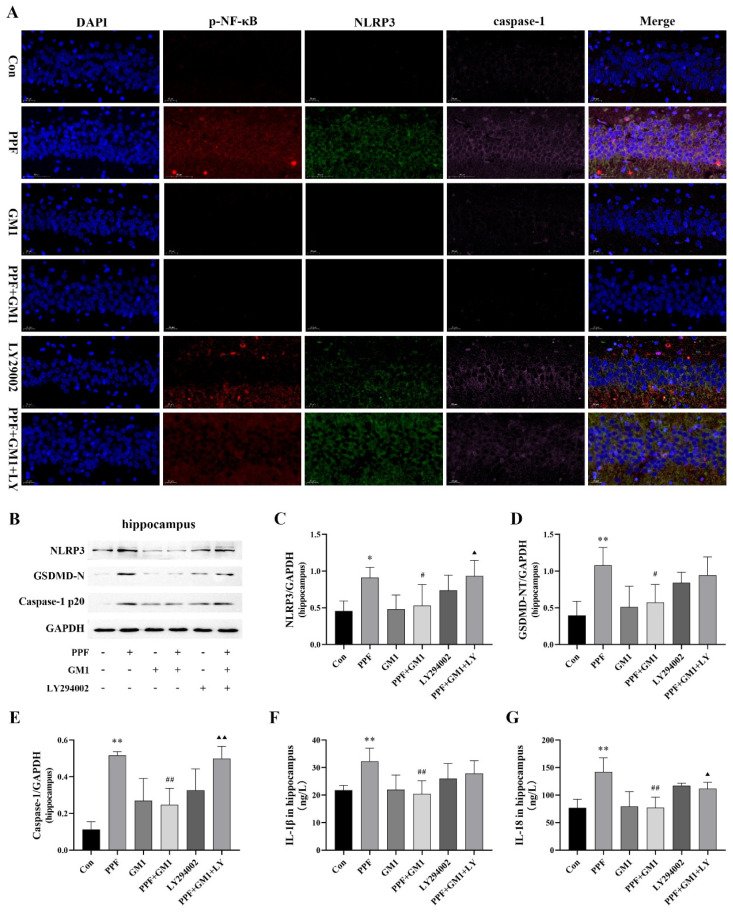
GM1 attenuates propofol-induced pyroptosis and inflammation in the hippocampus of young rats. (**A**) Immunofluorescence images of the CA1 region (NF-κB/NLRP3/caspase-1 triple labeling); scale bar: 20 μm. (**B**–**E**) Immunoblot images and corresponding quantification of NLRP3, p20, and GSDMD-N protein in the hippocampus. (**F**,**G**) Content of inflammatory cytokines in the hippocampus ((**B**–**E**), n = 3; (**F**–**G**), n = 6). * *p* < 0.05, ** *p* < 0.01 vs. Con, ^#^ *p* < 0.05, ^##^ *p* < 0.01 vs. PPF, ^▲^ *p* < 0.05, and ^▲▲^ *p* < 0.01 vs. PPF + GM1.

The impact of GM1 on inflammation and pyroptosis in the hippocampus following propofol treatment was assessed via Western blotting and ELISA kits. We observed an upregulation of pyroptosis proteins (NLRP3, p20, and GSDMD-N) as well as increased concentrations of IL-1β and IL-18 following propofol treatment. Nevertheless, a GM1 pretreatment caused a significant reduction in pyroptosis-related proteins and inflammatory cytokines, although this effect of GM1 was reversed upon treatment with LY294002 (Figure 4B–G). Collectively, these findings suggested that GM1 might alleviate propofol-related pyroptosis in the hippocampus. Furthermore, we utilized a triple-label immunofluorescence staining technique to examine the co-localization of p-NF-κB, NLRP3, and caspase-1 p20 in hippocampal tissue. These results revealed that propofol exposure increased the protein expression of p-NF-κB, NLRP3, and caspase-1 in the hippocampus (CA1 region) of developing rats. Notably, a GM1 treatment markedly diminished the activation of both NF-κB and the NLRP3 inflammasome (Figure 4A). However, LY294002 effectively negated the regulatory effects of GM1. These findings further suggested potential links between PI3K/AKT/NF-κB signaling and the neuroprotective effects of GM1.

### 2.4. Effect of GM1 on the Survival Rate of Propofol-Administered PC12 Cells

**Figure 5:**
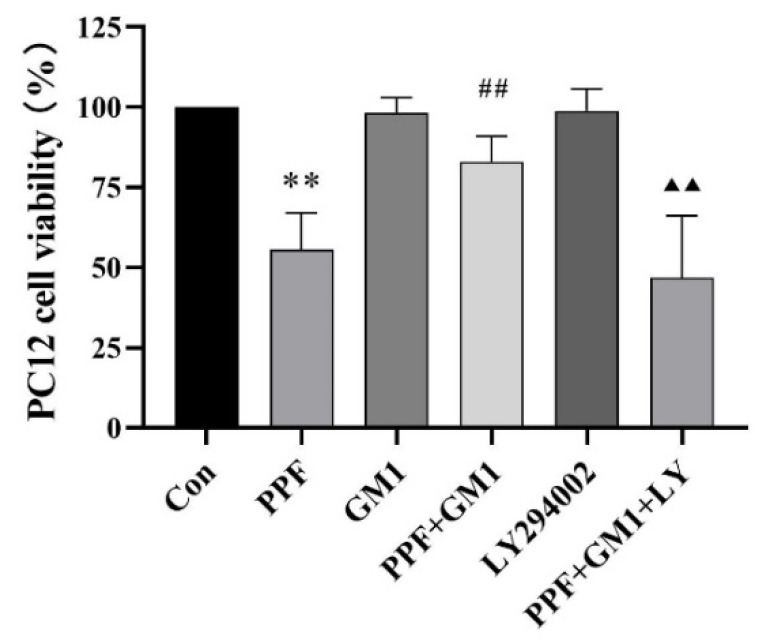
Impact of GM1 on propofol-induced cell viability in PC12 cells (n = 6). ** *p* < 0.01 vs. Con, ^##^ *p* < 0.01 vs. PPF, and ^▲▲^ *p* < 0.01 vs. PPF + GM1.

To further determine the mechanism of GM1’s neuroprotective effects, PC12 cell lines were used for in vitro experiments. Subsequently, a CCK-8 analysis was conducted to assess cell viability after a GM1 treatment. Interestingly, a significant reduction in the cell survival rate (55.59%) was found in the PPF group following 6 h of a 300 μM propofol treatment compared with the Con group (*p* < 0.01). Notably, a pretreatment with GM1 effectively mitigated propofol-induced cell death, while LY294002 counteracted the effect of GM1 in the LY + GM1 + PPF group (Figure 5).

### 2.5. Impact of GM1 on Propofol-Induced PC12 Cell Death via the PI3K/AKT/NF-κB Pathway

**Figure 6:**
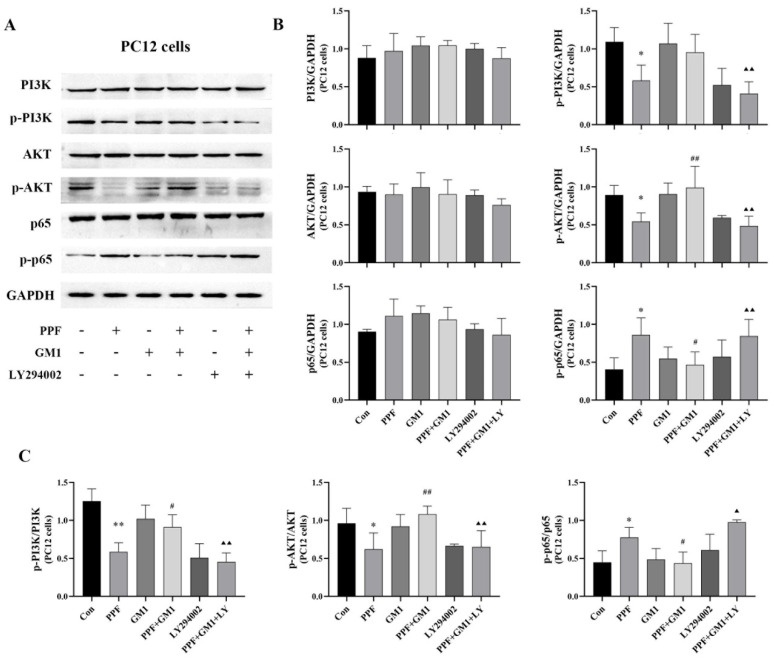
Regulation of the PI3K/AKT/NF-κB pathway by GM1 in PC12 cells following propofol exposure. (**A**) Immunoblots and representative bands of PI3K/AKT/NF-κB proteins. (**B**,**C**) Corresponding quantification analyses of PI3K, p-PI3K, AKT, p-AKT, p65, p-p65, p-PI3K/PI3K, p-AKT/AKT, and p-p65/p65 in the hippocampus (n = 3). * *p* < 0.05, ** *p* < 0.01 vs. Con, ^#^ *p* < 0.05, ^##^ *p* < 0.01 vs. PPF, ^▲^ *p* < 0.05, and ^▲▲^ *p* < 0.01 vs. PPF + GM1.

Western blotting was performed for a quantitative determination of phosphorylated and non-phosphorylated forms of proteins in this pathway. As shown in Figure 6, a GM1 pretreatment significantly improved the propofol-induced decrease in PI3K/AKT/NF-κB signaling in PC12 cells. The protein levels of p-PI3K and p-AKT, the p-PI3K/PI3K ratio, and the p-AKT/AKT ratio were notably upregulated (*p* < 0.01), while the protein content of p-p65 and the p-p65/p65 ratio were remarkably downregulated following a GM1 pretreatment (*p* < 0.01). Interestingly, LY294002 reversed the effect of GM1 in the PPF + GM1 + LY group. Overall, these findings suggested that the PI3K/AKT/NF-κB signaling cascade might be involved in GM1-mediated neuroprotection against propofol-induced neurotoxicity.

### 2.6. GM1 Inhibits NLRP3/Caspase-1 and Propofol-Induced Pyroptosis in PC12 Cells

**Figure 7:**
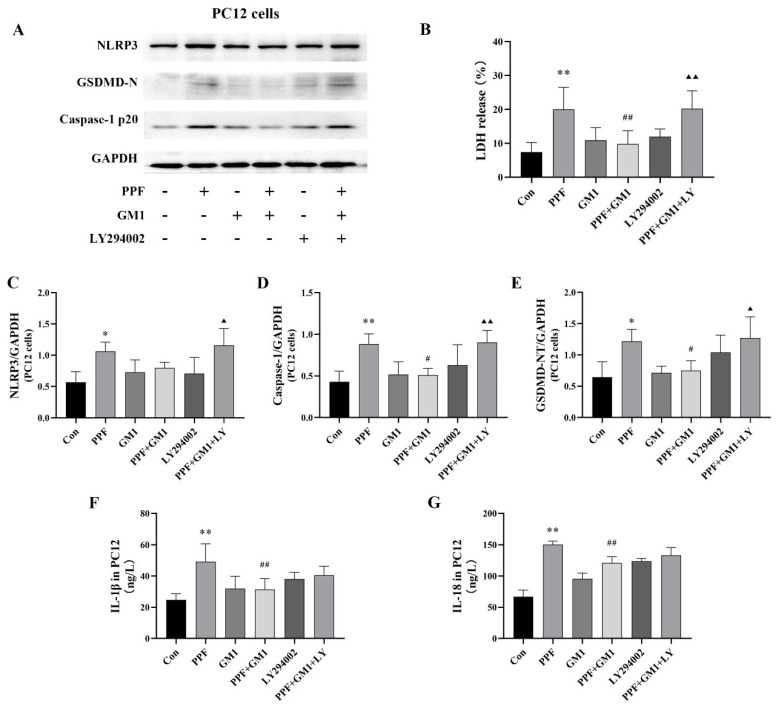
GM1 downregulates propofol-induced pyroptosis and inflammation in vitro. (**A**) Immunoblots and representative bands of NLRP3, p20, and GSDMD-N. (**B**) Concentrations of LDH release in PC12 cells. Corresponding quantification of NLRP3 (**C**), p20 (**D**), and GSDMD-N (**E**). (**F**,**G**) Pro-inflammatory cytokine levels in PC12 cells ((**A**–**E**), n = 3; (**F**,**G**), n = 6). * *p* < 0.05, ** *p* < 0.01 vs. Con, ^#^ *p* < 0.05, ^##^ *p* < 0.01 vs. PPF, ^▲^ *p* < 0.05, and ^▲▲^ *p* < 0.01 vs. PPF + GM1.

The release of pyroptosis-related pro-inflammatory cytokines and LDH was detected in PC12 cells. Our results, as shown in Figure 7, revealed that a pretreatment with GM1 led to reduced LDH release and neuroinflammation levels (IL-1β and IL-18) as well as downregulated pyroptosis-associated proteins (NLRP3, p20, and GSDMD-N) in PC12 cells. Notably, a pretreatment with LY294002 resulted in a contrasting trend between the LY + GM1 + PPF group and the GM1 + PPF group. These findings suggested that GM1 might attenuate propofol-induced pyroptosis through the PI3K/AKT/NF-κB cascade in PC12 cells.

## 3. Discussion

Propofol is frequently utilized for pediatric induction and maintenance anesthesia during surgical procedures as well as sedation in the ICU. However, the use of propofol in these situations is off-label, and the FDA only approves propofol for children over 2 months old for maintenance anesthesia and over 3 years old for induction anesthesia [26], raising concerns about its safety when used for pediatric anesthesia. Preclinical research has demonstrated that propofol can trigger developmental neurotoxicity across animal and cell models [27]. For example, propofol exposure causes immature hippocampal neuron injury and neural stem cell injury [28,29]. The promotion of cell death in immature neurons of propofol in rodents [30] and nonhuman primates (NHPs) has been demonstrated in several studies [8]. Our study explored propofol-induced nerve injury in young rats: PND 7 rats were given five consecutive doses of propofol (initial dose of 40 mg/kg, followed by 20 mg/kg) through an intraperitoneal injection with an interval of 60 min. In addition, propofol caused hippocampal injury and long-term behavioral impairments in PND 7 rats, mainly manifested as (1) the loss and disarrangement of CA1 and CA3 and (2) cognitive function deficits in the WMW test after adulthood.

The NF-κB signaling cascade is pivotal in cell survival, proliferation, differentiation, apoptosis, inflammation, and the immune response [31,32]. Phosphorylated NF-κB (p-p65) serves as a critical activator of downstream inflammatory signals. Under physiological conditions, NF-κB forms complexes with IκB (e.g., IKBα) and remains sequestered in the cytoplasm [33]. Pro-inflammatory mediators can activate IκB kinases (IKKs), resulting in IκB phosphorylation and degradation. Subsequently, the dissociation of the NF-κB complex allows free NF-κB to enter the nucleus and regulate downstream gene transcription [34]. Additionally, the phosphorylation of NF-κB facilitates the activation of the NLRP3 complex, subsequently triggering the expression of pro-IL-1β [35]. The inflammasome typically consists of pattern recognition receptors (PRRs; sensor proteins), link protein-ASC, and caspase-family proteins (e.g., caspase-1/4/5/11). Bacteria, viruses, and specific disease processes can activate intracellular PRRs. These PRRs can identify pattern-associated molecular patterns (PAMPs) and damage-associated molecular patterns (DAMPs). Ultimately, this induces the self-cleavage of caspase and the subsequent pore formation of GSDMD, leading to pyroptosis [36]. Previous research demonstrated that propofol activated NF-κB/NLRP3 signaling in a postoperative cognitive dysfunction (POCD) model in aged rats [37]. Interestingly, we observed that continuous propofol exposure led to NF-κB activation as well as NLRP3 assembly and pyroptosis in developing rats and PC12 cells through the increased phosphorylation of p-p65, the upregulation of pyroptosis-related protein levels, and increased concentrations of neuroinflammatory factors. Our preliminary findings suggested that NF-κB/NLRP3 pathway activation contributed to propofol-induced hippocampal injury and neuroinflammation. Regulating this process may be beneficial for the development of effective therapeutic strategies.

Therefore, to further investigate the potential mechanism and protective drugs in PPF-induced neurotoxicity in developing rats, we selected GM1 as a candidate therapeutic drug. GM1, widely present in the cell membrane, is extremely important in neural development and its deficiency can affect normal brain development [38]. Exogenous GM1 rapidly passes through the blood–brain barrier with high efficacy. Moreover, we found that GM1 did not affect the anesthetic effect of propofol. Currently, GM1 is widely used as a neuroprotective agent in various CNS diseases such as Parkinson’s disease (PD), Alzheimer’s disease (AD), and strokes [21]. In addition, GM1 also exerts a certain therapeutic effect against ischemic neurological damage by increasing its expression in the cerebral cortex and downregulating the expression of the NMDA receptor subunit NMDAR1, thereby maintaining normal neural function in middle cerebral artery occlusions (MCAOs) [39,40]. Previous studies by us and Meng et al. showed that exogenous GM1 significantly improved ketamine-induced hippocampal injury and cognitive deficits in developing rats [41,42]. In this study, we observed that a GM1 pretreatment mitigated hippocampal damage (CA1 and CA3 regions) and performance in the WMW test in young rats after propofol exposure. Concurrently, a GM1 pretreatment downregulated the NF-κB/NLRP3-related pyroptosis pathway and neuroinflammation in the hippocampus. PC12 cells were selected for in vitro studies, and we revealed that GM1 alleviated NF-κB pathway activation and pyroptosis induced by propofol. Overall, our findings indicated the potential protective effect of GM1 against propofol-related hippocampal injury during development.

The PI3K/AKT signaling cascade is pivotal in the regulation of diverse cellular functions, including cell proliferation and survival [43]. Here, we found that propofol increased p-PI3K and p-Akt levels in vivo and in vitro. These findings suggest a potential link between the neuroprotective impact of GM1 and PI3K/AKT pathway signaling. To further clarify the potential mechanism of GM1, LY294002 (a specific PI3K inhibitor) was used in this study. The results revealed that LY294002 negated the effect of GM1 on the PI3K/Akt cascade. Additionally, LY294002 reversed the protective effect of GM1 against propofol-induced developmental neurotoxicity. Importantly, the PI3K/AKT signaling cascade is closely associated with inflammation, and NF-κB serves as a downstream regulator of the PI3K/Akt cascade [44,45]. Following the elimination of GM1’s regulatory effect on the PI3K/AKT signaling cascade by LY294002, we observed the activation of the NF-κB pathway, which was accompanied by increased NLRP3-associated pyroptosis and neuroinflammation. Collectively, these preclinical findings suggested that GM1 might exert protective effects against propofol-induced developmental neurotoxicity by upregulating the PI3K/Akt pathway, inhibiting the NF-κB pathway, and alleviating pyroptosis in the hippocampus of developing rats. Nonetheless, the process of translating these findings from animal models into human medical clinical practice is currently hindered by numerous factors and remains profoundly uncertain, as follows, (1) whether our results are applicable to different development stages or other animals and (2) whether the variance in the exposure time of propofol has an influence on the neuroprotective effect of GM1 as well as (3) the difficulty in conducting clinical studies on the neuroprotective effects of GM1.

The limitations of this study included its small sample size and exclusive focus on rats, necessitating future research with larger sample sizes and the inclusion of other animal species to comprehensively assess the neuroprotective effects of GM1. In future research, it will be essential to conduct additional types of behavioral experiments and long-term observations to evaluate the impact of GM1 on cognitive function and its neuroprotective effects against propofol-induced developmental neurotoxicity. Furthermore, future research should explore the effects of GM1 on other pathways through the PI3K/AKT/NF-κB pathway (such as mitochondrial damage and oxidative stress) to better clarify the neuroprotective mechanisms of GM1.

## 4. Materials and Methods

### 4.1. Animals

Sprague Dawley (SD) rats at postnatal day 7 (PND 7; Changsheng Biotechnology, Liaoning, China) were housed in an environment with suitable temperature and humidity levels and a standard 12 h dark/light cycle.

### 4.2. Propofol Exposure and Drug Treatment

Neurotoxicity caused by propofol in PND 7 rats was generated using a previously documented protocol [46,47]. Each group consisted of 25 mice, with a total of 6 groups assigned. (1) The control rats (Con) received 0.9% sodium chloride via an intraperitoneal injection. (2) The propofol rats (PPF) were continuously exposed to propofol five times with a one-hour interval between each dose. The first dosage was 40 mg/kg, followed by four doses of 20 mg/kg. (3) The GM1 treatment group (GM1) received 10 mg/kg of GM1 (Qilu Pharmaceutical Co., Ltd., Shandong, China). (4) The GM1 + PPF treatment group (GM1 + PPF) was given 10 mg/kg of GM1 prior to propofol. (5) The LY294002 treatment group (LY294002) received LY294002 (10 mg/kg, MCE, Monmouth Junction, NJ, USA). (6) In the LY + GM1 + PPF treatment group, GM1 and LY294002 were injected 30 min before propofol administration. Each group received five doses of propofol or sodium chloride every 60 min. The rats in the treatment group intraperitoneally received GM1, LY294002, or NS 30 min before propofol administration. Each injection was administered at a volume of 0.1 mL. One hour after the last treatment, samples were gathered when the rats were euthanized. Tissue samples were obtained for the subsequent studies. Each group of 10 rats was raised until PND 30 for the testing of learning and memory abilities.

### 4.3. Nissl Staining Analysis

Whole-brain specimens were preserved in 4% paraformaldehyde for fixation overnight, followed by embedding in paraffin and sectioning with a microtome to obtain 5 μm slices. The slices were deparaffinized and transparent, and Nissl staining was applied. Histological images of hippocampal tissue were collected using a microscope (Leica, Wetzlar, Germany) by observers blinded to the experimental groups; morphological changes in the regions of CA1 and CA3 were assessed and cell counts were performed at a 400× magnification.

### 4.4. Morris Water Maze (MWM) Assay

We performed an MWM assay to assess cognitive ability in PND 30 rats (n = 10). The MWM assay was conducted according to our previous study [48]. In summary, the experiment comprised the following two phases: five days of training trials, followed by exploratory trials on day 6. During the training trials, rats were randomly released into the pool to locate a fixed platform. The test ended once the rat reached the fixed platform. In the exploratory trials, the fixed platform was thrown away. Rats were positioned at random locations and permitted to swim in the pool for 90 s to assess their spatial memory.

### 4.5. In Vitro Experiment and Propofol Exposure

Rat adrenal pheochromocytoma cells (PC12 cells) were sourced from the Clinical Veterinary Medicine Laboratory, Northeast Agricultural University (Hei Longjiang Province, Harbin, China). The PC12 cells were grown in DMEM (Meilunbio, Dalian, China) with 10% fetal bovine serum (FBS) and a penicillin–streptomycin–amphotericin-B Solution (Beyotime, Shanghai, China) at 37 °C in an environment of 5% CO_2_. The complete medium alone, GM1 (100 μM), LY294002 (20 μM), or GM1 (100 μM) plus LY294002 (20 μM) were applied, respectively, to PC12 cells for 30 min and then continuously exposed to propofol (300 μM) for 6 h.

### 4.6. CCK-8 Test

A commercialized kit (Biomake, Beijing, China) was used for the cell viability assay. The absorbance at 450 nm was measured for each treatment group, and the survival rate of PC12 cells was calculated.

### 4.7. Lactate Dehydrogenase Release Analysis

Based on a previous report [48], the colorimetric assay for lactate dehydrogenase (LDH) release rate detection was conducted utilizing a test kit (C0016, Beyotime) as per the provided instructions.

### 4.8. Immunofluorescence Assay

For the immunofluorescence (IF) study, five-micron-thick whole-brain tissue sections were floated on water maintained at 40 °C to achieve flattening. Subsequently, the sections were carefully retrieved using slides and placed in an oven set at 60 °C for fixation. Whole-brain tissue slices underwent antigen retrieval using a citrate buffer, followed by washing with PBS. Subsequently, goat serum was applied to the slices at room temperature for blocking (30 min). A primary antibody (NF-κB p-p65, 1:100, WanLei; NLRP3, 1:100, WanLei; caspase-1, 1:100, ABclonal) was then applied, followed by incubation overnight at 4 °C. Moreover, appropriately labeled enzyme-conjugated secondary antibodies were applied to the tissue slices for 90 min, followed by DAPI staining to visualize the nucleus. Images were obtained using a confocal microscope (Nikon Eclipse C1, Tokyo, Japan).

### 4.9. Western Blotting Analysis

The protein samples were treated with a loading buffer for 5X-SDS-PAGE and added to SDS-PAGE gel (10% or 12%) for separation. The blots were transferred to nitrocellulose (NC) membranes. Skimmed milk was used for blocking. Subsequently, the specific primary antibodies (PI3K, 1:1000, WanLei; p-PI3K, 1:1000, Affinity; AKT, 1:1000, WanLei; p-AKT, WanLei; caspase-1, ABclonal; NLRP3, ABclonal; NF-κB, WanLei; p-NF-κB, WanLei; GSDMD, 1:1000, ABCAM; GAPDH, 1:1000, ABclonal) were incubated overnight at 4 °C. Membranes were analyzed using an ECL Kit (Dakewe Biotech Co., Ltd., Shenzhen, China) following HRP-conjugated secondary antibody incubation (1:3000) at room temperature. The results were analyzed using Image J software (1.8 version), and all experiments were conducted in triplicate to ensure statistical reliability.

### 4.10. ELISA Test

Changes in IL-1β and IL-18 were detected using commercial ELISA kits (Jiangsu Jingmei Co., Ltd., Yancheng, China) following the instructions provided by the manufacturer.

### 4.11. Statistical Analysis

The data were presented as the mean ± standard deviation (SD). The statistical analysis involved a one-way analysis of variance (ANOVA) followed by Tukey’s post hoc test. The MWM results were assessed using a repeated two-way ANOVA followed by the LSD test. A significance level of *p* < 0.05 was established.

## 5. Conclusions

This study offers novel perspectives on the potential of GM1 to address propofol-induced neurotoxicity during developmental stages. Our findings demonstrated that GM1 exerted a neuroprotective effect and ameliorated propofol-induced neuroinflammation and cognitive dysfunction via the modulation of the PI3K/AKT/NF-κB signaling cascade.

## Data Availability

All the data used to support the findings during this study are included within the article.
